# Knockdown resistance (*kdr*) mutations in the Japanese encephalitis virus vector *Culex tritaeniorhynchus* from the Republic of Korea

**DOI:** 10.1186/s41182-026-00927-5

**Published:** 2026-03-24

**Authors:** Jiseung Jeon, Sunwoo Hwang, Kwang Shik Choi

**Affiliations:** 1 Department of Biology, College of Natural Sciences, Kyungpook National University, Daegu, 41566 Republic of Korea; 2 School of Life Sciences, BK21 FOUR KNU Creative BioResearch Group, Kyungpook National University, Daegu, 41566 Republic of Korea

**Keywords:** *Culex tritaeniorhynchus*, Pyrethroid insecticide, Knockdown resistance, Republic of Korea

## Abstract

**Background:**

*Culex tritaeniorhynchus* is the primary vector of Japanese encephalitis virus (JEV), a disease of global public health significance, and is widely distributed across Africa, Asia, Australia, and Europe. Despite its epidemiological importance as a JEV vector, studies on insecticide resistance in *Cx. tritaeniorhynchus* populations from the Republic of Korea (ROK) remain extremely limited. This study aimed to investigate the spatial and temporal occurrence of knockdown resistance (*kdr*) mutations in *Cx. tritaeniorhynchus* populations from the ROK. Additionally, the association between phenotypic resistance to pyrethroid insecticides and *kdr* alleles was examined.

**Methods:**

*Culex tritaeniorhynchus* specimens were collected from eight locations across the ROK to assess the occurrence of *kdr* mutations. Additionally, WHO tube bioassays were conducted using the pyrethroid insecticide deltamethrin to assess phenotypic resistance and its association with *kdr* mutations.

**Results:**

The *kdr* mutation 1014F was detected in all surveyed populations, and the frequency of the 1014F allele remained relatively stable during the peak abundance period of *Cx. tritaeniorhynchus* from July to September. Bioassay results further indicated that *Cx. tritaeniorhynchus* populations in the ROK exhibit resistance to pyrethroid insecticides. However, no clear association was detected between the presence of the 1014F mutation and pyrethroid-resistant phenotypes.

**Conclusions:**

These findings indicate the need for further investigations into insecticide resistance in *Cx. tritaeniorhynchus* to support effective vector control and mitigate the risk of JEV transmission. Moreover, the results suggest that pyrethroid resistance in *Cx. tritaeniorhynchus* may not be driven solely by *kdr* mutations, indicating the potential involvement of additional resistance mechanisms.

**Supplementary Information:**

The online version contains supplementary material available at 10.1186/s41182-026-00927-5.

## Background

*Culex tritaeniorhynchus* Giles is a primary vector of Japanese encephalitis virus (JEV) and is widely distributed worldwide [1]. Since the introduction of vaccination programmes in the 1970s, approximately 20–30 cases of JEV have consistently been reported annually in the Republic of Korea (ROK) [2]. Globally, an estimated 100,000 cases occur each year, with the majority reported from India and Southeast Asia [3]. Additionally, recent evidence suggesting the potential role of *Cx. tritaeniorhynchus* in the transmission of lumpy skin disease virus has further heightened interest in this species as a disease vector [4]. It is typically associated with rural environments and is reported to reach particularly high densities in rice paddies and around cattle sheds [5, 6]. Given the intensive concentration of agricultural and livestock production within a relatively small land area in the ROK, *Cx. tritaeniorhynchus*, which commonly inhabits paddy fields and livestock facilities, may be frequently exposed to a wide range of insecticides owing to its ecological characteristics [7].

Mosquito populations that are frequently exposed to insecticides experience strong selection pressure, which can drive the evolution and spread of insecticide resistance. To date, several resistance mechanisms have been described, such as target-site mutations, reduced cuticular penetration (penetration resistance), enhanced metabolic detoxification (metabolic resistance), sequestration-based resistance, and behavioural avoidance [8, 9]. The emergence and spread of resistance in disease vectors can compromise the effectiveness of control interventions and pose a significant threat to public health.

In the ROK, several insecticide classes, including neonicotinoids, benzoylureas, and organophosphates, are used for vector control. Among these, pyrethroids are reported to be the most widely used for mosquito control [10] because of their high insecticidal efficacy and comparatively low toxicity to humans [11]. However, the emergence of knockdown resistance (*kdr*) mutations in the voltage-gated sodium channel gene, along with cross-resistance, is increasingly recognised as a major limitation of pyrethroid-based control strategies [12–14]. In the ROK, *kdr* mutations associated with pyrethroid resistance have been investigated in several mosquito taxa, and *kdr* variants (1014F, 1014C, and 1014S) have been reported in *Anopheles* mosquitoes and the *Cx. pipiens* complex [15–17].

*Kdr* mutations in *Cx. tritaeniorhynchus* have previously been documented in China, which is geographically close to the ROK [18–20]. More recently, the *kdr* (1014F) mutation has also been detected in *Cx. tritaeniorhynchus* populations in the ROK [21, 22]. However, studies conducted in the ROK have provided limited insights into the relationship between *kdr* mutations and phenotypic resistance to pyrethroid insecticides. Yoo et al. [23] confirmed resistance to pyrethroids such as deltamethrin and cypermethrin through bioassays using field-collected *Cx. tritaeniorhynchus* larvae. However, as with other studies from the ROK, the association between resistance phenotypes and the presence of *kdr* mutations was not examined.

The potential presence of cryptic species within East Asian populations of *Cx. tritaeniorhynchus* has been repeatedly proposed [20, 24–28]. Mitochondrial DNA analyses further suggest that this taxon can be divided into two types, the Continental type (Ct-C) and the Japanese type (Ct-J) [24, 27, 28]. Because cryptic taxa may differ in insecticide susceptibility and resistance mechanisms among lineages, lineage-specific studies are essential for developing effective control strategies [29–31]. Moreover, unlike regions such as China and Taiwan, where both types have been reported to co-occur [20, 25, 26], *Cx. tritaeniorhynchus* populations in the ROK are reported to be currently dominated by the Ct-J type [27]. This may provide a comparatively favourable setting for investigating insecticide resistance in a single type.

To address these gaps in the literature, this study investigated the prevalence and geographic distribution of *kdr* mutations in *Cx. tritaeniorhynchus* populations from the ROK and examined the association between phenotypic resistance to pyrethroid insecticides and *kdr* alleles. The findings are expected to help address existing knowledge gaps regarding the relationship between *kdr* alleles and pyrethroid resistance in the ROK and to inform the development of more effective control strategies for *Cx. tritaeniorhynchus* to reduce JEV transmission.

## Methods

### Sample collection and identification

**Fig. 1:**
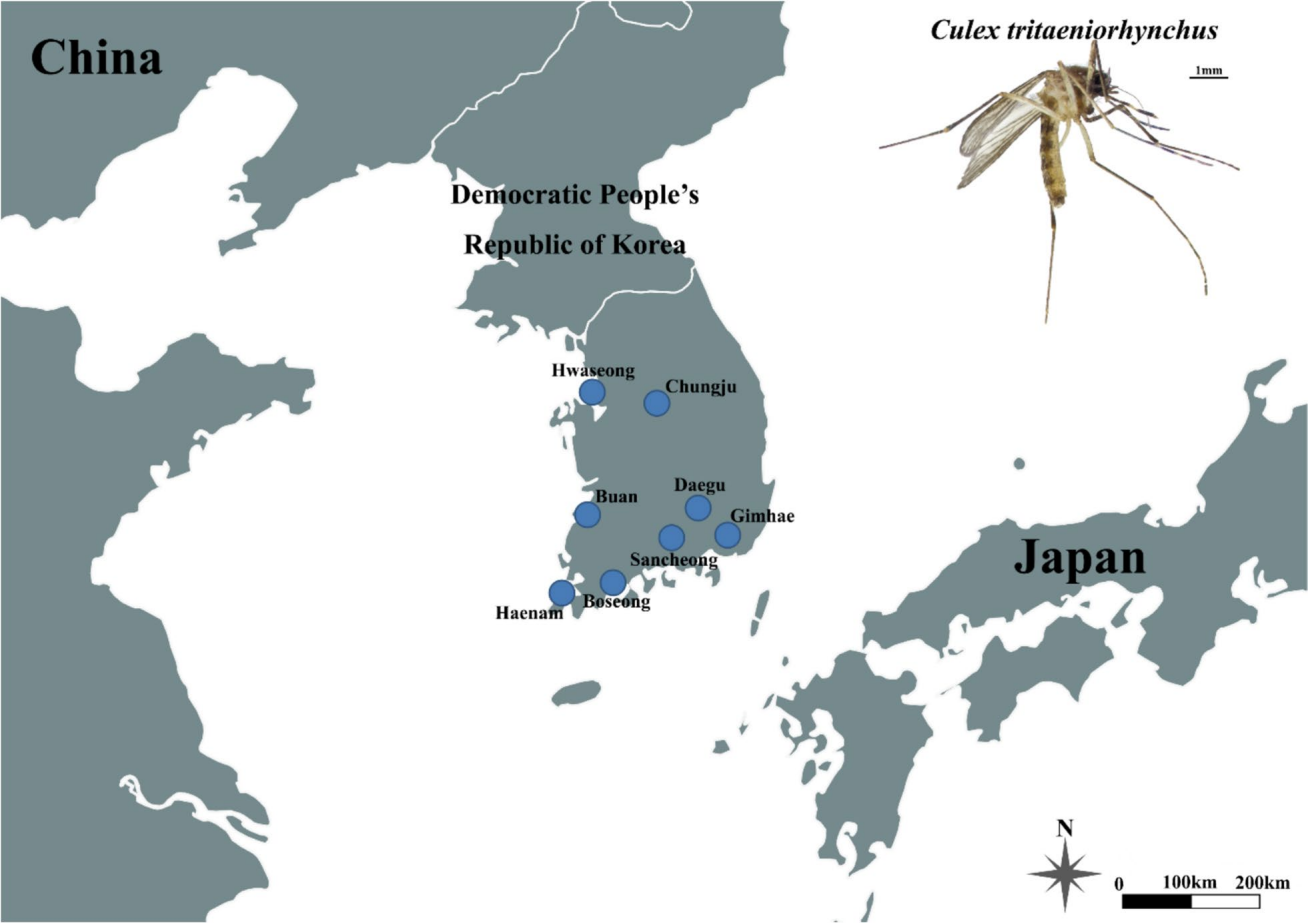
Collection sites of *Culex tritaeniorhynchus* used in this study. Detailed information on sampling locations is provided in Supplementary File 1. The map was generated using QGIS v3.26.3 (https://www.qgis.org/ko/site)

Adult *Cx. tritaeniorhynchus* were collected between July and September 2024 from cattle sheds at eight locations across the ROK. Sampling sites were selected based on the species’ primary habitats (Fig. 1). Collections were conducted using black-light traps (BT Global, Seongnam, ROK) with dry ice as a supplemental attractant. Collected mosquitoes were transported to Kyungpook National University (Daegu, ROK) and stored at −20 °C until species identification to minimise specimen damage. *Cx. tritaeniorhynchus* was distinguished from other mosquito species using standard morphological identification keys [32]. Following confirmation, genomic DNA was extracted from individual specimens using the Clear-S™ Quick DNA Extraction Kit (InVirusTech, Gwangju, ROK).

Molecular identification was performed to discriminate between the two *Cx. tritaeniorhynchus* types (Ct-J and Ct-C) currently recognised in East Asia. A fragment of the mitochondrial cytochrome *c* oxidase subunit I (*COI*) gene was amplified through polymerase chain reaction (PCR) using universal primer pairs (LCO1490: 5′-GGTCAAATCATAAAGATATTGG-3′; HCO2198: 5′-TAAACTTCAGGGTGACCAAAAAATCA-3′) [33]. The resulting amplicons were subjected to restriction enzyme digestion targeting a type-specific single nucleotide polymorphism, and type assignment was based on the PCR–RFLP profiles (Ct-J: 475 bp + 244 bp; Ct-C: 719 bp) [28]. Because Ct-C individuals have been reported at very low frequencies (< 2%) [27]. In the ROK, only Ct-J specimens, which are currently dominant in the country, were included in the analyses.

### Pyrethroid insecticide susceptibility bioassay

To obtain mosquitoes for insecticide susceptibility bioassays, blood-fed female *Cx. tritaeniorhynchus* were collected on four occasions throughout September 2025 from cattle sheds in Daegu, ROK (Fig. 1). Collections were conducted using a black-light trap (BT Global, Seongnam, ROK) and an aspirator. Field-collected females were maintained under standard insectary conditions (27 ± 1 °C, 75% relative humidity, and a 16 h:8 h light:dark photoperiod) to induce oviposition.

Bioassays were conducted using unfed F1 females aged 2–3 days post-emergence derived from oviposited eggs from nine egg batches. Insecticide susceptibility testing was performed in accordance with World Health Organization (WHO) guidelines [34]. To compare mortality across concentrations, deltamethrin-impregnated papers at 0.03% and 0.05% were used. A total of 50 unfed females were assigned to the control group and exposed to insecticide-free papers. For each insecticide concentration, 100 females were exposed to the corresponding impregnated paper for 1 h, and mortality was recorded 24 h post-exposure. Knockdown and surviving individuals were subjected to genomic DNA extraction using the Clear-S™ Quick DNA Extraction Kit (InVirusTech, Gwangju, ROK). Type identity was confirmed using the PCR–RFLP assay described above, and the specimens were used for molecular analyses.

### Detection of* kdr* mutations

PCR assays were performed to screen for *kdr* mutations in specimens obtained from the bioassays and from populations collected across the ROK. Amplification was performed using the primer pair developed by Lee et al. [22] (Cxt_vgsc-F: 5′-GACCTGCCACGGTGGAACT-3′; Cxt_vgsc-R: 5′-GATCTTGTTGTTTCGTTGTCG-3′). The PCR reaction mixture (25.0 μL total volume) contained 1 × PCR buffer, 0.2 mM dNTPs, 0.4 μM of each primer, 0.5 U of HotStart Taq DNA polymerase (TaKaRa, Shiga, Japan), and 1 μL of extracted genomic DNA. Cycling conditions consisted of an initial denaturation at 94 °C for 5 min; 30 cycles of 94 °C for 30 s, 58 °C for 30 s, and 72 °C for 1 min; and a final extension at 72 °C for 5 min. PCR products were visualised through electrophoresis on a 1.5% agarose gel, yielding amplicons of approximately 1000–1100 bp. Successfully amplified products were subjected to Sanger dideoxy sequencing using the PCR primers (Macrogen, Daejeon, ROK) (Supplementary File 1: Fig S1). Consensus sequences generated in this study were deposited in NCBI GenBank (accession numbers: PX578863–PX578864). For bioassay-derived specimens, all individuals exhibiting a resistant phenotype were analysed for *kdr* mutations, along with a subset of susceptible individuals (*n* = 30) for comparison.

### Data analysis

To assess seasonal variation in allele frequencies, the frequencies of the 1014F and 1014L alleles were calculated for each sampling period. Specimens collected on days 1–15 of each month were classified as the first sampling round, whereas those collected on days 16–30 were classified as the second sampling round. Allele frequencies were compared across sampling periods from the second round of July to the second round of September.

Exact tests for Hardy–Weinberg equilibrium (HWE) were performed using Genepop v4.7 [35]. Differences in resistance-allele frequencies among sampling sites, as well as associations between *kdr* mutations and insecticide-resistance phenotypes, were evaluated using Fisher’s exact test and Chi-square tests. Odds ratios (ORs) and 95% confidence intervals (CIs) were calculated, and statistical significance was set at *p* < 0.05. All statistical analyses and visualisations were conducted in R v4.3.3 (https://www.R-project.org/) using the ggplot2 package v3.5.2 [36].

## Results

### Pyrethroid insecticide susceptibility of *Culex tritaeniorhynchus*

**Fig. 2:**
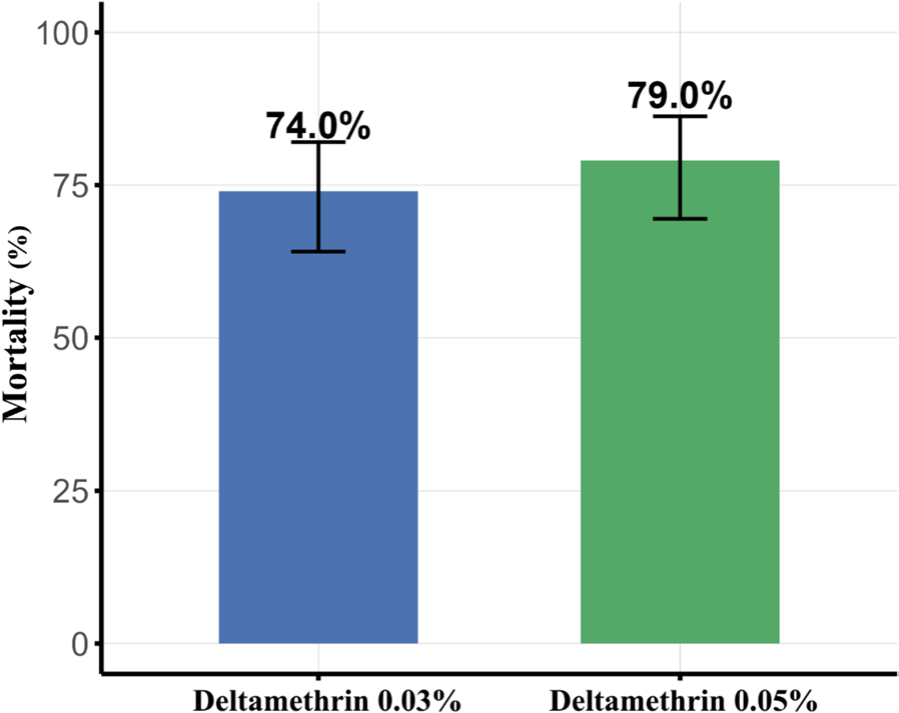
Results of WHO tube bioassays using two different concentrations of deltamethrin. Error bars indicate 95% confidence intervals (95% CI)

Molecular identification confirmed that all individuals used in the experiments belonged to the Ct-J type. WHO tube bioassays indicated that *Cx. tritaeniorhynchus* collected from Daegu, ROK, in 2025 exhibited resistance to pyrethroid insecticides. Twenty-four hours after exposure to insecticide-impregnated papers, mortality was 74% (95% CI 64.1–82.0%) at 0.03% deltamethrin and 79% (95% CI 69.5–86.2%) at 0.05% deltamethrin (Fig. 2). Although mortality was higher at the higher concentration, the difference was not statistically significant (Fisher’s exact test, *p* = 0.51).

The results of *kdr* genotyping for resistant and susceptible individuals are summarised in Table 1. Among the 26 individuals showing a resistant phenotype following exposure to 0.03% deltamethrin, 5 were homozygous resistant (1014F/1014F), 3 were heterozygous (1014L/1014F), and 18 were homozygous susceptible (1014L/1014L). Of the 21 resistant individuals exposed to 0.05% deltamethrin, 7 were 1014F/1014F, 1 was 1014L/1014F, and 13 were 1014L/1014L. As shown in Table 1, the proportion of the homozygous resistant genotype (1014F/1014F) was comparatively higher among individuals with a resistant phenotype, whereas the heterozygous genotype (1014L/1014F) was more frequently observed among susceptible individuals; however, these differences were not statistically significant (Table 1). Overall, no significant association was detected between the 1014F allele and resistance in the 0.03% deltamethrin group (OR = 0.45, 95% CI 0.13–1.51, *p* = 0.18). In the 0.05% deltamethrin group, the 1014F allele frequency was higher in resistant individuals; however, the association was not statistically significant (OR = 3.00, 95% CI 0.70–14.28, *p* = 0.11).

**Table 1:** Relationship between the frequency of the 1014F allele and resistance phenotypes under two different deltamethrin concentrations

Insecticide	Phenotype	N	Genotype			Allele frequency		OR	95% CI	*p*-value
			1014L/1014L	1014L/1014F	1014F/1014F	1014L	1014F			
Deltamethrin (0.03%)	Resistant	26	18	3	5	0.75	0.25	0.45	0.13 – 1.51	0.18
	Susceptible	30	15	14	1	0.73	0.27			
Deltamethrin (0.05%)	Resistant	21	13	1	7	0.64	0.36	3.00	0.70 – 14.28	0.11
	Susceptible	30	25	3	2	0.88	0.12			

### Distribution and frequency of* kdr* mutations in *Culex tritaeniorhynchus*

**Fig. 3:**
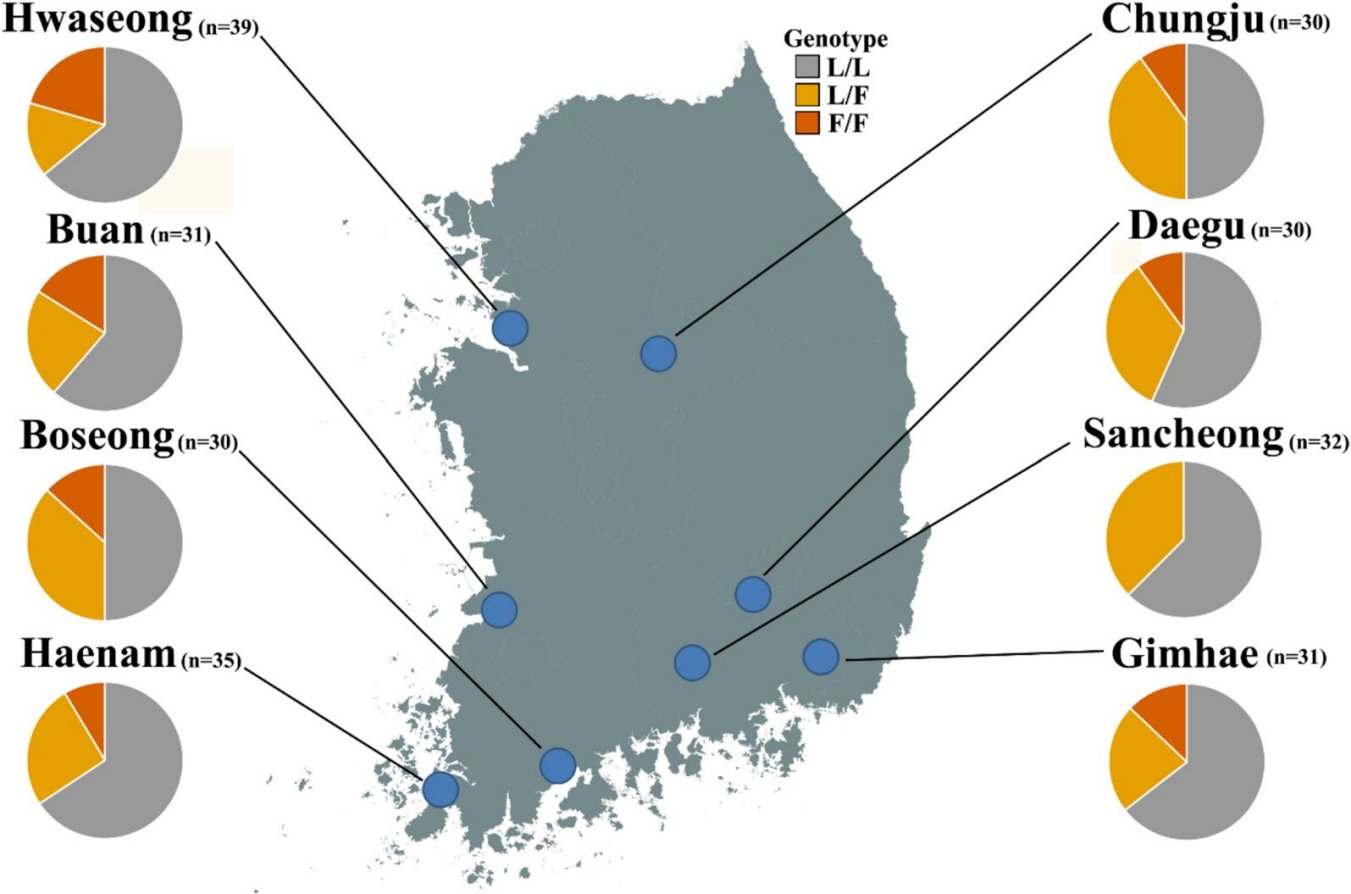
Geographic distribution of the 1014F and 1014L alleles across sampling regions

Among 258 *Cx. tritaeniorhynchus* (Ct-J) specimens collected from eight sites in the ROK in 2024, the frequency of the 1014F *kdr* allele was assessed. The 1014F allele was detected at all sampling locations (Fig. 3). The lowest 1014F allele frequency was observed in Sancheong (1014F = 0.19). Among the 32 individuals analysed from Sancheong, no homozygous resistant (1014F/1014F) individuals were detected. The highest 1014F allele frequency was recorded in Boseong (*n* = 30, 1014F = 0.32) (Table 2). Overall, 1014F allele frequencies were similar across all sampled sites in the ROK (0.19–0.32). To assess whether 1014F allele frequencies differed among populations, a Chi-square test of homogeneity based on allele counts was performed. No significant differences were detected among sampling sites (*χ*^2^ = 3.8019, df = 7, *p* = 0.80). HWE tests indicated deviations from HWE in Hwaseong, Buan, and Gimhae, consistent with heterozygote deficiency (*p* < 0.05), whereas all other populations aligned with HWE expectations (Table 2).

**Table 2:** Frequencies of the 1014F *kdr* allele in *Culex tritaeniorhynchus* collected in the Republic of Korea

Sites	N*	Genotype			Allele frequency		HWE test (*p*–value)
		1014L/1014L	1014L/1014F	1014F/1014F	1014L	1014F	Probability test**
Hwaseong	39	25	6	8	0.71	0.29	0.01
Chungju	30	15	12	3	0.70	0.30	1.00
Daegu	30	17	10	3	0.73	0.27	0.38
Buan	31	19	7	5	0.73	0.27	0.02
Sancheong	32	20	12	0	0.81	0.19	0.55
Boseong	30	15	11	4	0.68	0.32	0.41
Gimhae	31	20	7	4	0.76	0.24	0.04
Haenam	35	23	9	3	0.79	0.21	0.16
Total	258	154	74	30	0.74	0.26	

^*^N = total number of individuals;

^**^Populations not in conformity to Hardy–Weinberg equilibrium at *p* ≤ 0.05

**Fig. 4:**
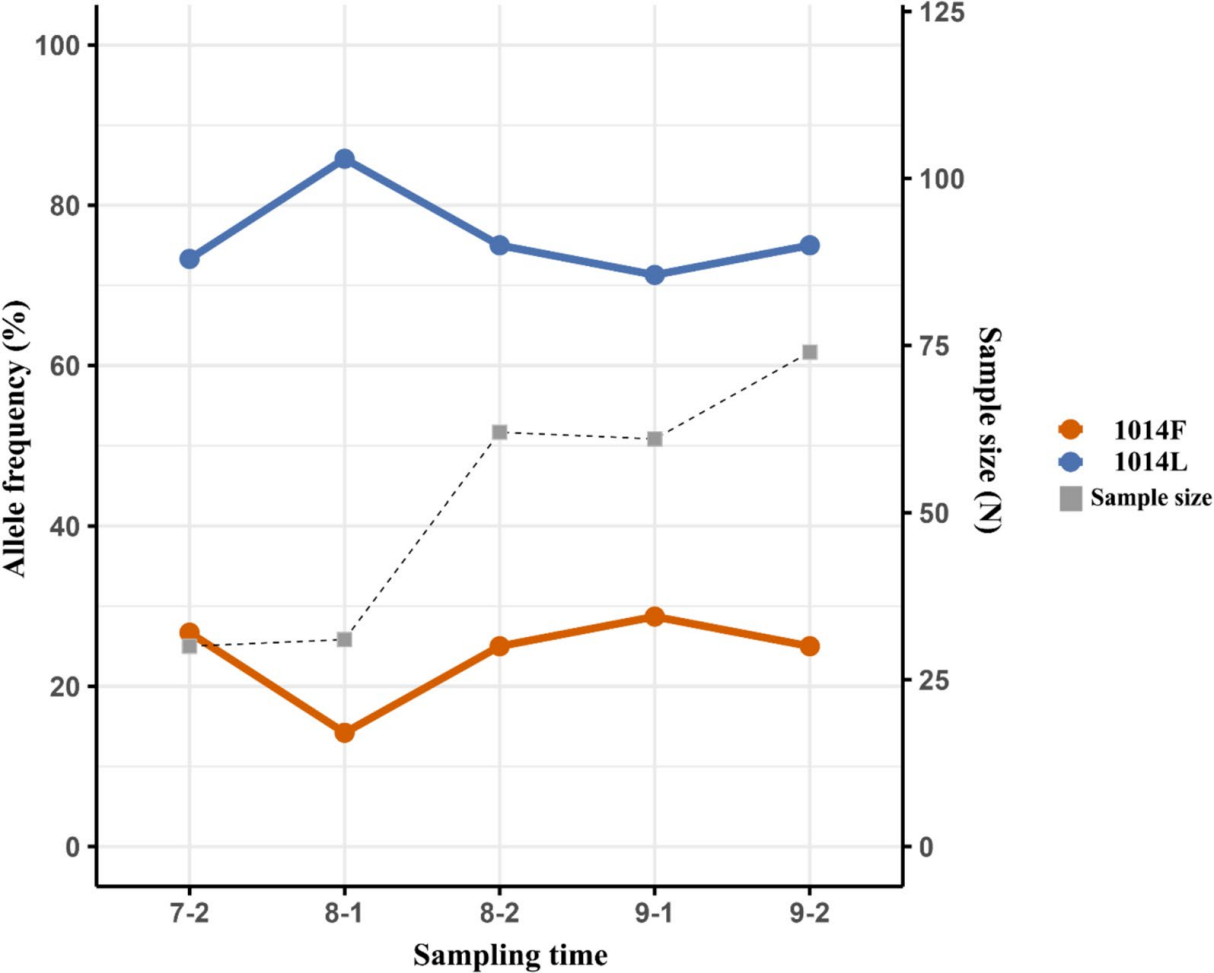
Temporal changes in the proportions of the 1014F and 1014L alleles by sampling period. Days 1–15 of each month were classified as the first sampling period, whereas days 16–30 were classified as the second sampling period

Seasonal changes in the 1014F allele frequency were assessed by comparing allele frequencies across sampling periods (Fig. 4). Although a fluctuating pattern was observed, the 1014F allele frequency did not differ significantly among time points (*χ*^2^ = 4.622, df = 4, *p* = 0.32). From the first sampling period (second round of July) to the final sampling period (second round of September), the 1014F allele frequencies were 0.27 (*n* = 30) in July (round 2), 0.14 (*n* = 31) in August (round 1), 0.25 (*n* = 62) in August (round 2), 0.29 (*n* = 61) in September (round 1), and 0.25 (*n* = 74) in September (round 2).

## Discussion

Yoo et al. [23] previously reported pyrethroid resistance in *Cx. tritaeniorhynchus*; however, that study was conducted using larvae rather than adults. Furthermore, the presence of the 1014F *kdr* allele has already been documented in *Cx. tritaeniorhynchus* from selected locations in the ROK [21, 22]. However, these earlier studies did not evaluate the association between the 1014F allele and phenotypic resistance and were limited by a small number of sampling sites. The present study was conducted to help address these gaps. Although the WHO tube bioassays confirmed that *Cx. tritaeniorhynchus* is resistant to pyrethroid insecticides, a clear association between resistance phenotypes and the 1014F allele was not observed. Notably, through nationwide sampling, we demonstrate for the first time that the 1014F allele is already present across diverse regions of the ROK. This study also has limitations. Bioassays were performed using mosquitoes collected only from Daegu; therefore, insecticide susceptibility patterns may not fully represent those of other regional populations. Future studies should extend phenotypic resistance testing to populations from additional regions beyond those examined here. For more robust comparative interpretation of bioassay outcomes, future work should include a well-characterised laboratory susceptible reference strain to provide a consistent baseline for comparison. However, a standardised susceptible strain is not currently available for this species in our laboratory or, to our knowledge, within the ROK, underscoring the need to establish and maintain such a reference colony for improved cross-study comparability. Moreover, because a trend toward higher susceptibility was observed at the higher deltamethrin concentration, further comparative assays using a broader range of deltamethrin concentrations, as well as other pyrethroid compounds, will be necessary. In this regard, our findings provide baseline data to inform and guide subsequent studies.

Across all sampling sites in the present study, the frequency of the 1014F allele (0.19–0.32) showed little geographic variation, and no marked seasonal shifts were observed (0.14–0.29). The relatively stable maintenance of the mutant allele frequency across multiple locations and time points may reflect broadly comparable insecticide-selection pressures irrespective of site or sampling period, along with ongoing gene flow among populations [37, 38]. Although pyrethroids are reportedly the most widely used insecticides for mosquito control in the ROK [10], applications are largely concentrated from spring to autumn, whereas usage declines sharply during winter, when vector activity and abundance decrease. The comparatively low 1014F allele frequency (approximately 0.2–0.3) may therefore be consistent with a fitness cost associated with *kdr* mutations [39, 40]. Such costs could limit the spread of the mutant allele under conditions of weak or intermittent selection. Accordingly, further studies are required to directly assess whether *kdr* mutations impose measurable fitness costs in *Cx. tritaeniorhynchus* populations. In addition, because the present analysis was limited to specimens collected between July and September, broader seasonal sampling will be necessary and may help to further test and validate this hypothesis.

Wu et al. [18] conducted bioassays of *Cx. tritaeniorhynchus* populations in China using deltamethrin, permethrin, and beta-cypermethrin, and reported a significant association between pyrethroid resistance and *kdr* allele frequency. By contrast, no clear association between pyrethroid resistance and the 1014F allele was observed in *Cx. tritaeniorhynchus* populations from Türkiye [41]. Consistent with the latter findings, our study did not detect a statistically significant association between the 1014F allele and resistance phenotypes. A mismatch between *kdr* genotypes and resistance phenotypes, and the recognition that *kdr* alone explains only part of the resistance phenotype, has been reported across several mosquito species [42, 43]. Moreover, an increasing number of novel loci associated with insecticide resistance continue to be identified [44–46]. Therefore, it is plausible that the extent to which *kdr* alleles predict resistance phenotypes varies among regions and populations, resulting in the observed discordance in *Cx. tritaeniorhynchus*. Recent research on *Cx. quinquefasciatus*, a primary vector of West Nile virus, supports this interpretation: strong deltamethrin resistance was observed when *kdr* mutations co-occurred with overexpression of cytochrome P450 genes [47].

Taken together, the available evidence suggests that although *kdr* mutations may contribute to pyrethroid resistance, they are unlikely to fully account for resistance phenotypes when considered in isolation [42]. In line with this interpretation, resistance in the bioassays was observed in some individuals carrying the homozygous susceptible genotype (1014L/1014L), supporting the involvement of additional resistance mechanisms beyond target-site insensitivity. However, metabolic resistance mechanisms were not directly assessed in the present study, and further investigations incorporating enzyme activity assays or synergist bioassays will be required [41]. The relatively stable frequencies of the 1014F allele observed across sites and sampling periods further indicate that *kdr* mutations alone are insufficient to drive the observed pyrethroid resistance phenotypes. If *kdr* mutations were the primary determinant of pyrethroid resistance, strong selection would be expected to drive pronounced spatial or temporal shifts in allele frequencies [48, 49]. However, the relatively consistent frequencies observed here suggest that, in addition to potential fitness costs, *kdr* contributes only partially to resistance, with other mechanisms, such as metabolic detoxification, likely playing important roles. Accordingly, future studies should investigate not only *kdr* mutations but also additional mechanisms underlying pyrethroid resistance in *Cx. tritaeniorhynchus*. Such studies will be essential for overcoming the limitations of pyrethroid-based control strategies and improving the effectiveness of vector-management strategies.

Among the two *Cx. tritaeniorhynchus* clades currently recognised in East Asia (Ct-C and Ct-J), the present study focused exclusively on the Ct-J type, which is dominant in the ROK. Ct-J also predominates in Japan, and intermittent introductions of Ct-C via wind-assisted dispersal have been suggested [24, 27]. In contrast, both clades are reported to co-occur in China and Taiwan [20, 25, 26]. To date, however, it remains unclear whether Ct-C and Ct-J differ in their insecticide-resistance mechanisms. Because our study characterised pyrethroid resistance and the occurrence of the 1014F *kdr* allele in Ct-J populations, complementary investigations focusing on Ct-C are warranted. Lineage-specific differences in resistance mechanisms among cryptic taxa have already been documented in *Anopheles* mosquitoes [30, 31, 50]. If resistance mechanisms differ between the two clades, examining whether these mechanisms, or resistance-associated alleles, can move between clades depending on reproductive compatibility and gene flow could provide valuable insights for improving vector-control strategies and understanding the evolutionary dynamics of insecticide resistance.

## Conclusion

This study aimed to clarify the relationship between insecticide-resistance phenotypes and genotypes in *Cx. tritaeniorhynchus*. Our findings indicate that *kdr* mutations alone are insufficient to fully explain phenotypic resistance to pyrethroid insecticides, suggesting that additional resistance mechanisms may be involved. Given that *Cx. tritaeniorhynchus* is a primary vector of JEV, continued investigation into insecticide resistance is essential for informing effective control strategies. In this context, our results provide important baseline data for future studies aimed at elucidating the mechanisms underlying insecticide resistance in *Cx. tritaeniorhynchus*.

## Supplementary Information

Supplementary Material 1.

## Data Availability

The datasets supporting the conclusions of this article are included within the article.

## Abbreviations

JEV: Japanese encephalitis virus
ROK: Republic of Korea
*Kdr*: Knockdown resistance
Ct-C: Continental type
Ct-J: Japanese type
PCR: Polymerase chain reaction
WHO: World Health Organization
HWE: Hardy–Weinberg equilibrium
