# Supplementary material for: Knockdown resistance (kdr) mutations in the Japanese encephalitis virus vector Culex tritaeniorhynchus from the Republic of Korea
Source: Trop Med Health. 2026 Mar 24;54:68. doi: 10.1186/s41182-026-00927-5 (PMC13064233; doi:10.1186/s41182-026-00927-5)
Supplement: Supplementary file 1 — Supplementary Material 1. [file 41182_2026_927_MOESM1_ESM.docx]

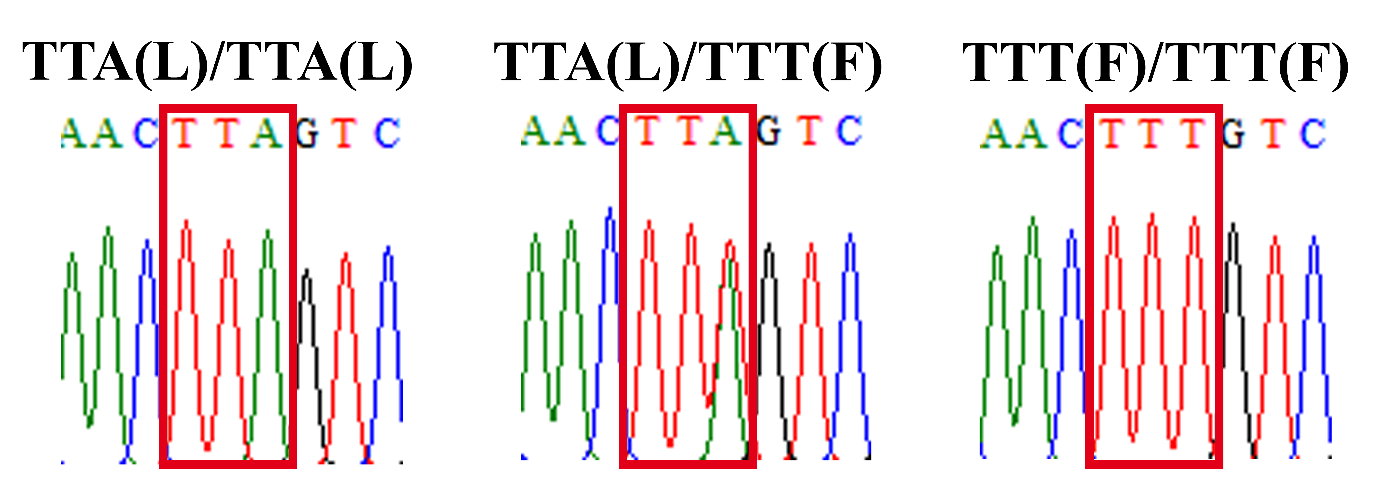


**Fig S1.** Chromatogram of the DNA sequencing of the kdr gene from Culex tritaeniorhynchus.  Homozygous susceptibility, TTA/TTA (L1014/ L1014). Homozygous resistance, TTA/TTT (1014L/ 1014F).  Homozygous resistance, TTT/TTT (1014F/ 1014F).

**Table S1.** Collection sites information used in this study

| Collection sites | Collection date | Coordinates |
| --- | --- | --- |
| Hwaseong | 2024.09.25 | 37°06'34"N, 126°47'28"E |
| Chungju | 2024.09.12 | 36°58'30"N, 127°46'29"E |
| Daegu | 2024.07.26 | 35°41'36"N, 128°24'02"E |
| Buan | 2024.09.08 | 35°36'42"N, 126°43'09"E |
| Gimhae | 2024.08.04 | 35°21'24"N, 128°51'03"E |
| Sancheong | 2024.08.25 | 35°19'31"N, 127°59'54"E |
| Boseong | 2024.08.25 | 34°46'01"N, 127°06'27"E |
| Haenam | 2024.09.17 | 34°38'16"N, 126°19'48"E |
